# Alveolar Socket Preservation with Different Autologous Graft Materials: Preliminary Results of a Multicenter Pilot Study in Human

**DOI:** 10.3390/ma13051153

**Published:** 2020-03-05

**Authors:** Elio Minetti, Edoardo Giacometti, Ugo Gambardella, Marcello Contessi, Andrea Ballini, Gaetano Marenzi, Martin Celko, Filiberto Mastrangelo

**Affiliations:** 1 Private Practice, 20100 Milan, Italy; elio.minetti@gmail.com; 2 Private Practice, 10121 Torino, Italy; edoagiac@libero.it; 3 Private Practice, 24068 Seriate, Italy; ugo.gambardella@gmail.com; 4 Private Practice, 34121 Trieste, Italy; contegiallots@hotmail.it; 5 Department of Biosciences, Biotechnologies and Biopharmaceutics, Campus Universitario “Ernesto Quagliariello”, University of Bari “Aldo Moro”, 70125 Bari, Italy; andrea.ballini@uniba.it; 6 Department of Basic Medical Sciences, Neurosciences and Sense Organs, University of Bari Aldo Moro, 70125 Bari, Italy; 7 Department of Neurosciences, Reproductive and Odontostomatological Sciences, Federico II University, 80133 Naples, Italy; gaetano.marenzi@gmail.com; 8 Private Practice, 500 02 Hradec Kralove, Czech Republic; doktorcorporal@seznam.cz; 9 Department of Clinical and Experimental Medicine, University of Foggia, 71122 Foggia, Italy

**Keywords:** bone substitute materials, TT transformer, autologous tooth graft, bone tissue regeneration

## Abstract

Background: The histological and histomorphometrical results were evaluated between vital whole and non-vital endodontically treated teeth used as autologous grafts in post-extractive socket preservation procedures. Methods: Twenty-eight patients (average age 51.79 ± 5.97 years) with post-extractive defects were enrolled in five dentistry centers. All patients were divided into two groups: with whole teeth (Group 1) and teeth with endodontical root canal therapy (Group 2). The extracted teeth were processed with the Tooth Transformer device to obtain a demineralized and granulated graft material used with a resorbable collagen membrane for socket preservation. After four months, 32 bone biopsies were obtained for histological, histomorphometric, and statistical analysis. Results: During the bone healing period, no infection signs were observed. Nineteen biopsies in group 1 and 13 biopsies in group 2 were detected. The histological analysis showed neither inflammatory nor infective reaction in both groups. Autologous grafts surrounded by new bone were observed in all samples and, at high magnification, partially resorbed dentin and enamel structures were detected. No gutta-percha or cement was identified. Small non-statistically significant differences between the groups, in total bone volume (BV), autologous graft residual, and vital bone percentage were detected. Conclusions: The study showed that the TT Transformer grafts were capable of producing new vital bone in socket preservation procedures. The histomorphometric results showed no statistical differences comparing whole and endodontically treated teeth in bone regeneration. Further studies will be carried out in order to understand the advantages of the autologous graft materials obtained from the tooth compared with the current biomaterials in bone regeneration treatments.

## 1. Introduction

After tooth loss, natural bone remodeling is carried out, with volumetric alveolar bone reduction (vertical 1.67–2.03 mm and horizontal 3.87 mm) [1] and hard and soft tissue remodeling found to be higher during the first year [2,3]. To prevent volumetric bone loss, various surgical techniques were suggested with or without the use of graft materials and resorbable or non-resorbable membranes [4,5].

Fresh or demineralized freeze-dried human, animal (xenograft), and artificial (allograft) materials, used alone or combined, were studied in the literature in order to reduce the volumetric contraction of hard tissues (average bone loss of 0.36 mm horizontally and 0.58 mm vertically) [4,6].

For a long time, autogenous bone was considered the “gold standard” for its osteogenic, osteoconductive and osteoinductive properties; however it may have some problems, such as donor site morbidity and surgery, limited availability, and, in some cases, a high resorption rate [7]. For these reasons, in the last 10 years, great effort was applied to developing a large number of biomaterials, with rapid or slow reabsorption, used as scaffolds that show osteoconductive properties [8,9].

Many studies confirmed a similar composition of hydroxyapatite in the inorganic component and type 1 collagen, as well as other proteins in the organic component of bones, dentin, and enamel, although with different percentages [10,11,12]. In 1967, Bang et al. showed the osteoinduction potential features of demineralized dentin matrix [13,14] and, in 1991, Bessho et al., in an animal model, detected the presence of bone morphogenetic proteins (BMPs) in a human dentin matrix after a demineralization process [15]. In 2017, Rijal theorized how the dentin demineralization process of autologous extracted teeth allows better bone augmentation for the increased availability of BMPs [16]. Kim (2017) and Minamizato (2018) showed the efficacy of a chairside-prepared autologous partially demineralized dentin matrix for clinical bone regeneration procedures in human [17,18].

The aim of the present study was to evaluate the histological behavior of two different autologous graft materials from healthy whole vs. endodontically treated teeth, used in human as a demineralized graft material produced by the innovative TT Transformer medical device for a clinical alveolar socket preservation procedure.

## 2. Materials and Methods

Between February 2018 and October 2018, 28 patients (average age 51.79 ± 5.97 years) with 34 post-extractive defects and in good health condition were enrolled in five dental centers in Italy. All patients signed a written informed consent form before being included in the study, and they were assigned into two different groups: Group 1 (G1) with healthy whole teeth and Group 2 (G2) with teeth extracted for endodontic root canal treatment. After tooth extraction, all teeth were cleaned, separated, and automatically demineralized with the TT Transformer medical device.

All patients received the same alveolar socket preservation procedure using the autologous tooth as grafting material covered with a resorbable collagen membrane porcine pericardium (Bego oss®).

### 2.1. Inclusion Criteria

The study included patients over 18 years of age who needed a tooth extraction treatment, in good health condition (ASA-1 and ASA-2), and who were able to undergo dental surgical and restorative procedures. Tooth extractions were needed for trauma, caries, or periodontal diseases. The alveolar socket preservation procedures were requested in order to maintain the bone volume for dental implant rehabilitation after tooth extraction.

### 2.2. Exclusion Criteria

Pregnant subjects, patients with a history of allergies, tobacco use (within the last six months), diabetes, cancer, human immunodeficiency virus (HIV), bone or metabolic diseases, immunosuppressive agents, or use of systemic corticosteroids or intramuscular/intravenous bisphosphonates, and patients in radio or chemotherapy were excluded.

### 2.3. Preoperative Work-Up

Clinical and radiographic analysis with cone-beam computed tomography (CBCT, Planmeca ProMax 3DS Helsinki, Finland), periapical X-rays, or panoramic X-rays was performed. Two weeks before the oral surgery treatment, all patients received a professional oral hygiene session, and chlorhexidine 0.2% mouth rinses, twice a day for two weeks, were prescribed.

### 2.4. Surgical Procedures and Follow-Up

Antibiotic administration (2 g of amoxicillin/clavulanic acid in one solution 2 h before the extractions) was performed. The dimensions and the post-extractive socket morphology were recorded through direct measures. All extracted teeth were firstly cleaned using a diamond drill with abundant irrigation. Afterward, the G1 teeth (healthy whole) were cut into 5-mm-long samples.

In the G2 teeth (endodontic treatment) the filling materials (gutta-percha, composite, etc.) were firstly carefully removed and then cut in the same dimensions. All materials were inserted in the TT grinder device (TT Tooth Transformer srl. Milan, Italy) for automatic single-use demineralization procedures for 25 min.

Next, the bone defects were filled with a particulate demineralized dentin and enamel graft covered with absorbable membranes (Bego oss® Bremen, Germany). A second surgical stage was requested for the implant fixture placement at four months. Before the implant insertion, a bone biopsy was performed using trephine cylindrical drills graduated to indicate the depth (from 5 to 18 mm) with abundant irrigation using sterile saline.

### 2.5. Collection and Statistical Analysis of Data

Histological and morphometrical data were detected in accordance with the protocol recorded at the University of Chieti (ethical committee approval: request ID richhtnc4, protocol N°1869 12/12/2018, approved 17 verb 21.03.19 St.638 PI Perfetti). Data statistical analysis was carried out to obtain average values and to compare the behavior of the G1/G2 groups. Outcome measures of the exploratory study were analyzed with a *t*-test for paired samples for pre–post differences with time as the factor using Statistical Package for Social Sciences (SPSS for Windows, Version 11.5, Chicago, IL, USA) software, to detect significant differences between pre-test and post-test scores.

### 2.6. Histological Technique

The sample was dehydrated with a series of alcohol solutions of increasing concentration, and then fixed into methacrylic resin. After that, the sample was processed to obtain non-decalcified sections using a disc abrasion system (LS2 Remet, Bologna, Italy) and a diamond disc cutting system (Micromet Remet, Bologna, Italy) with a high speed in order to obtain a 200-μm-thick slide sample. With low abrasive paper, the sample was then abraded to progressively reduce the sample thickness to about 40–50 μm. Then, the samples were colored with basic fuchsin/blue toluidine and observed using light/polarized light microscopy. For histomorphometric measurements, the histological images obtained using the transmitted light microscope were digitized through a digital camera and analyzed by means of image analysis software IAS 2000; for each sample, the percentage of vital bone (VB%), the percentage of the remaining graft (Graft%), and the percentage of residual bone volume (BV%) were detected.

## 3. Results

Twenty-eight subjects (10 men and 18 women) of 51.79 years (±5.97) average age were enrolled for the research. Thirty-four teeth were extracted and used for alveolar socket preservation treatment. Twenty teeth in G1 and 14 teeth in the G2 group were included.

After all surgery treatments, no complications were shown, and 32 biopsies (19 in G1 and 13 in G2 group) were performed in second-stage surgery after four months of healing time. The histological analysis of the samples showed that dentin and enamel graft materials, partially resorbed, were surrounded and included in various new bone layers (Table 1, Table 2, Table 3 and Table 4).

**Figure 1:**
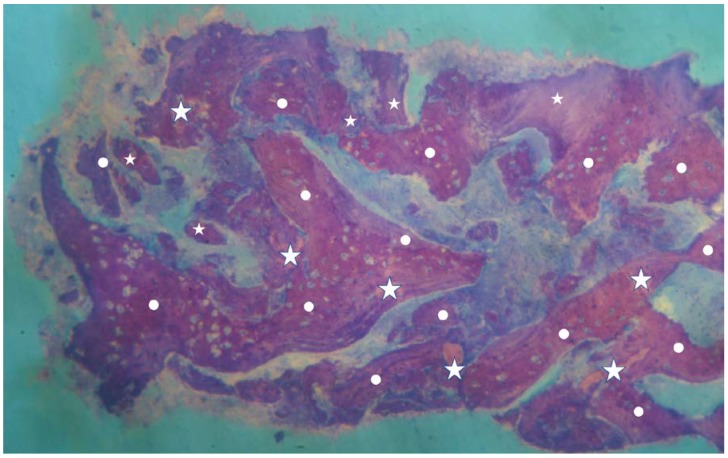
Overview of a biopsy at low magnification. Group 1: dentin matrix granules (indicated by white stars) originating from whole tooth completely surrounded by newly formed bone (indicated by white circles) are visible. No inflammatory or other adverse reaction is visible around the particles (magnification 8×; toluidine blue).

**Figure 2:**
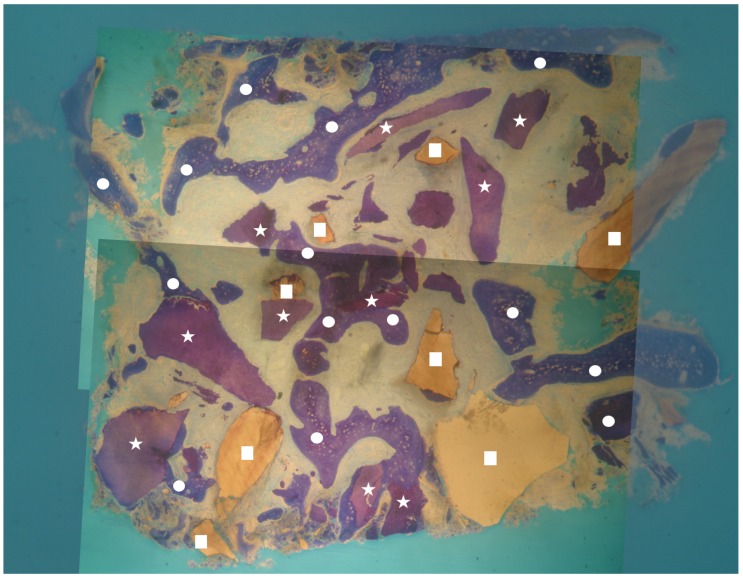
Overview of a biopsy at low magnification. Group 2: dentin matrix granules (indicated by white stars) and enamel granules (indicated by white squares) originating from endodontically treated tooth completely surrounded by newly formed bone (indicated by white circles) are visible. No inflammatory or other adverse reaction is visible around the particles (magnification 8×; toluidine blue).

**Figure 3:**
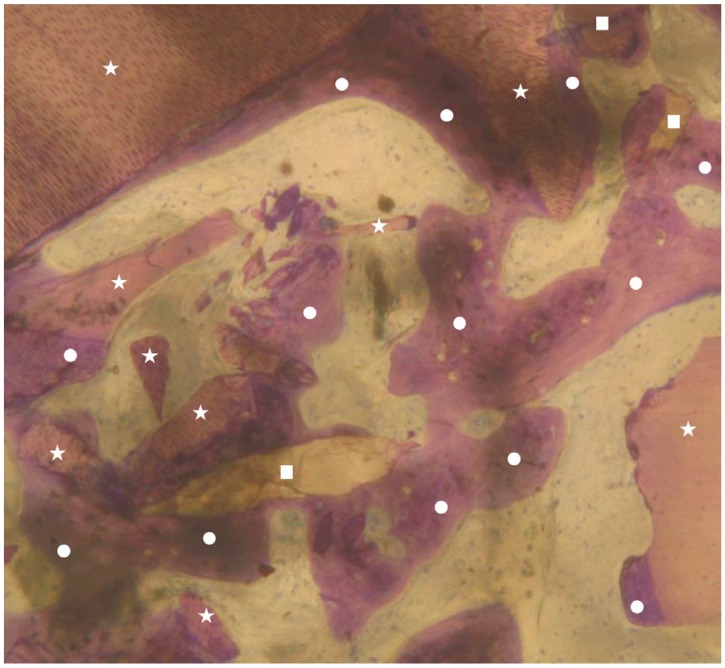
Group 1: newly formed bone trabeculae (indicated by white circles), dentin matrix graft particles (indicated by white stars), and enamel granules (indicated by white squares) are visible. It is possible to observe both granules (dentin and enamel) almost completely surrounded by new bone (indicated by white circles). No inflammatory or other adverse reaction is visible around the particles (magnification 25×; toluidine blue).

**Figure 4:**
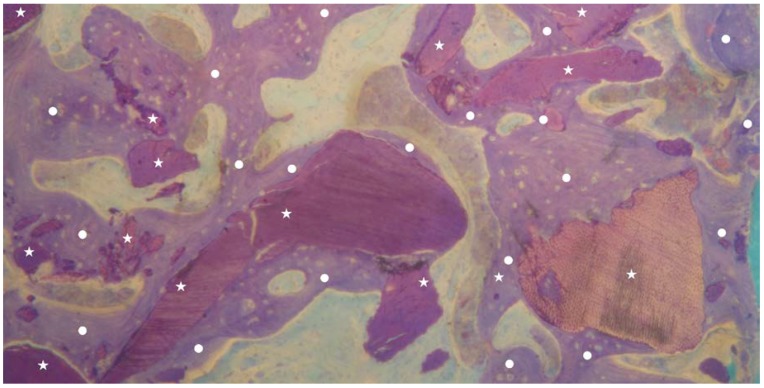
Group 2: newly formed bone trabeculae (indicated by white circles) and dentin matrix graft particles (indicated by white stars) are visible. It is possible to observe granules almost completely surrounded by new bone (indicated by white circles). No inflammatory or other adverse reaction is visible around the particles (magnification 25×; toluidine blue).

No inflammation signs were detected in all specimens (Figure 1 and Figure 2). No endodontic filling materials (gutta-percha, composite, cement, etc.) were detected in G2 samples (Figure 2). Histomorphometric analysis of the G1 biopsies showed a mean of 36.68% (±8.90%) for BV, 19.70% (±13.75%) for RG, and 20.78% (±13.29%) for VB (Figure 1 and Figure 3). In the G2 group, the histomorphometric analysis showed a mean of 39.16% (±11.51%) for BV, 17.39% (±7.09%) for RG, and 22.89% (±9.72%) for VB (Figure 2 and Figure 4). No statistical significance value (Table 5) was detected between the two groups.

## 4. Discussion

The knowledge of the physio-pathological processes following tooth extraction suggest constant three-dimensional bone reabsorption in height and thickness, which is higher in buccal vs. lingual–palatal regions [19,20].

In the last 15 years, several surgical procedures, involving intra- or extra-oral autologous bone or heterologous biomaterials, were proposed to limit these processes [6,8]. However, no studies showed only predictable benefits and responses [9,10,11,12].

As of today, the best results were shown in the use of autologous bone for its osseoconduction and osseoinduction characteristics [3,4]. However, all procedures required a second surgical site for bone harvesting or a double surgery treatment with increased discomfort of the patient [3,4]. To limit the discomfort, several biomaterials with slow or rapid reabsorption were suggested; however, all materials showed only osteoconductive capabilities [7,8].

From these scientific considerations, and from several studies of dental tissue embryology, the first aim of research should be to verify if the extracted tooth, currently considered waste material [21,22,23], could be used as graft material in alveolar socket preservation procedures [24]. Furthermore, the behavior of the endodontically treated tooth in bone regeneration procedures should be determined, as well as if the tooth, properly cleaned after endodontic treatment, could be used in these surgical procedures [25,26].

The results of the study confirmed the high biocompatibility of demineralized dental tissue used in socket preservation procedures. No inflammation signs or clinical failure were seen in all surgical procedures. No surgical sites showed difficulty in healing, and no resorbable membranes were discovered. No clinical or histological signs of inflammation or necrosis were detected in all sites or samples analyzed. All histological specimens showed no gutta-percha, composite, or cement filling materials. All histological results of demineralized autologous tooth materials showed a high value of vital bone around the grafts, capable of preventing volumetric bone loss in post-extracted alveolar sockets.

After alveolar socket preservation treatment using an autologous demineralized dentin/enamel graft material, the histomorphometric analysis showed total bone volume and vital bone percentages higher in Group 2 vs. Group 1, while a higher residual graft value was detected in Group 1 vs. Group 2. However, no statistically significant differences between the two groups were detected.

Furthermore, the extracted tooth was totally autogenous, with a dentin structure and composition very similar to bone. Dentin and enamel after TT Transformer treatment showed similar features to heterologous or synthetic bone substitutes on the market, whereas no expensive costs and no additional surgical procedures are required; it was also well accepted without further discomfort for the patient.

## 5. Conclusions

Several studies will be needed to know the real impact of this innovative technology on dental and maxillo-facial hard tissue regeneration therapy; however, the very promising results of our study show a high percentage of new vital bone around the residual graft material, suggesting that the autogenous demineralized tooth graft obtained by the TT Transformer medical device can be considered a feasible alternative to biomaterials currently used in human alveolar socket preservation procedures to promote bone healing in intraoral defects.

## Figures and Tables

**Table 1:** Demographic analysis of patients.

GROUP	AGE	Confidence Interval	Average Age	GENDER
Whole tooth—Group 1	55.31 ± 13.75	Min 47.83 Max 62.78	51.79 ± 5.97	11 F/2 M
Endodontical group—Group 2	48.27 ± 12.45	Min 41.97 Max 54.57		7F/8M
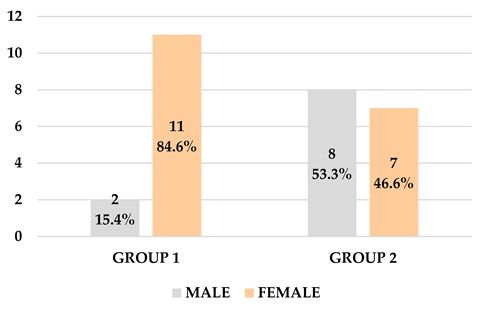				

**Table 2:** Number of teeth per patient.

**Group 1**		
1 Tooth	13	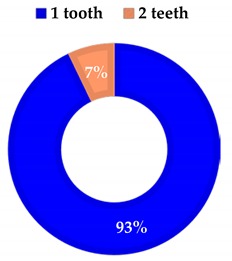
2 Teeth	1	
Average	1.07	
**Group 2**		
1 Tooth	11	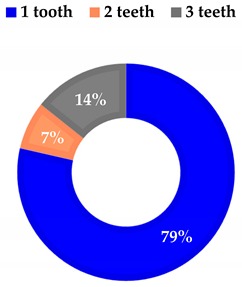
2 Teeth	1	
3 Teeth	2	
Average	1.4	

**Table 3:** Tooth analysis type and site.

**Group 1**		
**incisors**	3	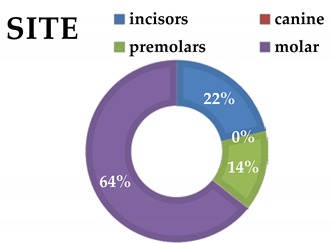
canine	0	
premolars	2	
molars	9	
**Group 2**		
incisors	3	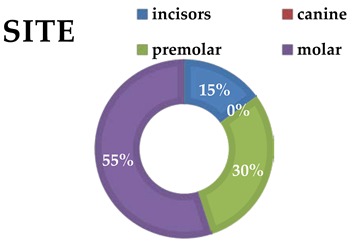
canine	0	
premolars	6	
molars	11	

**Table 4:** Etiology for each extraction.

	Prosthetic Failure	Infections
Group 1	9	6
Group 2	10	9
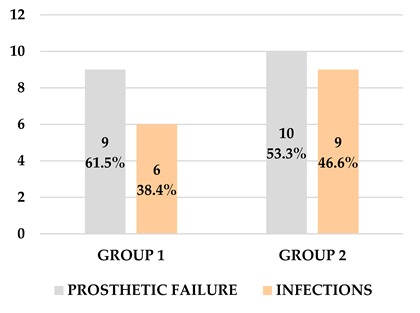		

**Table 5:** Statistical analysis (*p* > 0.005 denotes no statistical significance).

	BV Bone Volume		RG Residual Graft		VB Vital Bone	
Dataset	1	2	1	2	1	2
Sample Size	19	13	19	13	19	13
Average	36.6800	39.1600	19.7017	17.3983	20.7800	22.8900
Standard Deviation	8.9032	11.5104	13.7533	7.0935	13.2970	9.7245
T	0.6871		0.5536		0.4887	
Degree of Freedom	30		30		30	
*p*-value (significance level)	0.4973		0.5840		0.6286	
Analyze: *p* > 0.05 No statistical significance value *p* < 0.05 The hypothesis is wrong						
